# Conditioned stimulus effects on paired or alternative reinforcement depend on presentation duration: Implications for conceptualizations of craving

**DOI:** 10.3389/fnbeh.2022.958643

**Published:** 2022-08-04

**Authors:** Brett C. Ginsburg, Acacia Nawrocik-Madrid, Charles W. Schindler, R. J. Lamb

**Affiliations:** ^1^Department of Psychiatry and Behavioral Sciences, The University of Texas Health Science Center at San Antonio, San Antonio, TX, United States; ^2^Designer Drug Unit, Intramural Research Program, National Institute on Drug Abuse, Baltimore, MD, United States

**Keywords:** alcoholism, craving, relapse, stimulus control, conditioned suppression, choice, Pavlovian, operant

## Abstract

Conditioned stimuli (CS) associated with alcohol ingestion are thought to play a role in relapse by producing a craving that in turn increases motivation to drink which increases ethanol-seeking and disrupts other ongoing behavior. Alternatively, such CS may provide information indicating a likely increase in the density of the paired unconditioned stimulus and simultaneously elicit behavior that may be incompatible with other ongoing behavior, i.e., approach toward the CS. To explore these possibilities, rats were trained to respond for ethanol or food in two different components of the same session after which a light above the ethanol-lever was lighted twice during each component and each light presentation was followed by ethanol delivery. The duration of this CS was 10 s initially and then increased to 30 s, then to 100 s, and finally returned to 30 s. The change in responding for ethanol or food was compared to a matched period immediately preceding CS presentation. The CS presentation increased responding to ethanol, and this effect *increases* with longer CS presentations. In contrast, the CS presentation decreased responding to food, and this effect *decreases* with longer CS presentations. These results appear to support the informational account of CS action rather than simply a change in the motivation to seek and consume ethanol. This suggests that craving as it is commonly understood likely represents multiple behavioral processes, not simply increased desire for alcohol and that reports of craving likely reflect labeling based upon past experiences rather than a cause of future drug-taking.

## Introduction

In this manuscript, we report the results of an experiment varying the duration of a stimulus preceding ethanol delivery on responding reinforced by food in the first component of a multiple schedule and by ethanol in the second component. This experiment was occasioned by a popular conceptualization of craving and its role in relapse and excessive drug intake. In this worldview, craving is the result of Pavlovian conditioning and is a manifestation of increased motivation to take drugs. This increased motivation is thought to increase the probability of relapse and excessive drug intake both directly through increased motivation and indirectly by distracting the individual from other tasks that might compete with drug use. In other experiments, we have already addressed whether increases in behavior might result from increased motivation that result from Pavlovian conditioning (Lamb et al., 2016a,^2017^, ^2019^). In this experiment, we attempt to address whether increases in drug-seeking *and* decreases in other behavior during drug-paired stimuli are a result of these stimuli increasing motivation to consume drugs by examining whether drug-paired stimuli have comparable but opposing effects on behavior maintained by drug (increase) or an alternative reinforcement (decrease) across several different stimulus presentation durations.

There is ample evidence that at least under certain conditions, stimuli correlated with the delivery of the event that is reinforcing responding can increase that responding. For instance, food-paired stimuli can increase responding maintained by food. This has been shown for both animals responding to food (Lovibond, 1983) and in animals whose responding to food is in extinction (e.g., Estes, 1943). Similarly, ethanol-paired stimuli can increase responding to ethanol (Lamb et al., 2020), and ethanol- or cocaine-paired stimuli can increase responding to these drugs that are in extinction [Kruzich et al., 2001; Krank, 2003; Corbit and Janak, 2007, 2016; Krank et al., 2008; see Lamb et al. (2016c) for a review and critique of this literature].

Conversely, there is also ample evidence that stimuli paired either with significant negative events or significant positive events can disrupt ongoing behavior. Often, a stimulus paired with electric shock will disrupt food-maintained behavior [Estes and Skinner, 1941; Hunt and Brady, 1951; but see Waller and Waller (1963) for a counter-example]. Similarly, a stimulus paired with the delivery of food, water, or electrical brain stimulation will suppress responding maintained by food or water delivery (Azrin and Hake, 1969). Importantly, it has also been shown that cocaine-paired (Schindler et al., 2000), amphetamine-paired (Duncan et al., 1989; Watanabe, 1990), and pentobarbital-paired (Duncan, 1997) stimuli can disrupt food-maintained behavior. These increases or decreases in responding induced by paired stimuli may result from their effects on motivation, but other explanations are also possible.

Presumably, if both increased drug-seeking and disruption of other behavior result from increased motivation to take drugs then these should co-vary, i.e., disruption of other behavior should be positively correlated with increased drug-seeking. Thus, a manipulation that simultaneously changes the effectiveness of an ethanol-paired stimulus at increasing ethanol-maintained responding and decreasing food-maintained behavior might allow us to dissect whether these increases and decreases were resulting from the same mechanism, presumably motivation. One such manipulation is the duration of the paired stimulus. Stein et al. (1958) demonstrated that suppression of food-maintained responding by a shock-paired stimulus was greatest when the stimulus duration was short relative to the total session time without the stimulus. Henton and Brady (1970) showed that as the duration of a stimulus paired with food delivery increased, so too did the likelihood of increases in food-maintained responding during stimulus presentations. Meltzer and Brahlek (1970) also found that response increases were more likely with a longer stimulus duration and response suppression more likely with a shorter stimulus duration when the effects of a food-paired stimulus were studied on food-maintained behavior. Miczek and Grossman (1971) found that food-paired stimuli of shorter durations, but not a longer duration, suppressed food-maintained responding. Thus, it appears that short stimulus presentations are more likely to suppress responding, and relatively longer stimulus presentations are more likely to increase responding.

These findings argue that the suppression of behavior and the facilitation of behavior seen following the presentation of paired stimuli may not result from motivational changes *per se*, but rather from differences in the behavior elicited following presentations of stimuli differing in duration. However, these studies only looked at the effects of the paired stimulus on behavior maintained by a single event, yet it is the disruption of behavior other than that maintained by the (CS-paired) US that is hypothesized to result from increased motivation for the US. On the other hand, if the resulting effects of the CS on responding were a result of the information added to that context, then more nuanced results might be seen. The CS elicited goal approach decreases as CS length increases. CS also signal an increased density of US delivery. The first may disrupt ongoing behavior regardless of what is maintaining behavior. The latter may well increase behavior that is maintained by the US. Thus, behavior maintained by a reinforcer other than the US will be disrupted by shorter CS and relatively unaffected by longer CS. The response disruptive and response facilitating effects of short-duration CS may offset each other for behavior maintained by the US, while at longer CS, the response facilitating effects of the CS may be more apparent as the goal approach becomes less frequent. Therefore, the motivational and informational accounts of CS effects on responding make distinctly different predictions about what we should see as we manipulate the duration of the ethanol-paired CS and examine its effects on food- and ethanol-maintained behavior. The results of this experiment could provide further support for the notion that craving-induced facilitation of drug-seeking and disruption of other behavior both result from increased motivation to seek drugs. Alternatively, it could provide support for an informational account, and the idea that craving is a subjective effect representing a self-assessment of one’s likelihood of taking drugs when attempting not to take drugs [Tiffany, 1990; see Lamb et al. (1991) and Lamb and Henningfield (1994) for a discussion of subjective effects], as decreases in other behavior seen with short-duration CS and increases in drug-seeking seen with long-duration CS both increase the probability of future drug-taking by increasing the relative probability of drug-taking (see Lamb et al., 2016b; Lamb and Ginsburg, 2018).

People are said to crave a drink in two situations: The first is when stimuli associated with drinking increase their likelihood of wanting or seeking a drink, particularly when a drink might be unavailable or they are attempting not to drink. The second is when stimuli associated with drinking disrupt other ongoing behavior. Both effects are thought to be a result of stimuli associated with drinking increasing motivation to drink through Pavlovian conditioning. If this is the case, then manipulations that make stimuli predicting drink availability more effective at disrupting other ongoing behavior should also make stimuli more effective at increasing seeking an opportunity to drink; and conversely, manipulations that make stimuli more effective at increasing seeking an opportunity to drink should also make stimuli more effective at disrupting other behavior. Alternatively, if craving is simply a learned description of situations in which one’s behavior has been altered by stimuli associated with drinking, then we would not necessarily expect a positive correlation between the disruption of other behavior and an increase in behavior that might lead to a drink. In this experiment, we examine whether changes in the duration of the CS associated with ethanol delivery similarly changes the effectiveness of this CS at disrupting other behavior and increasing ethanol-seeking.

## Materials and methods

### Subjects

All procedures conducted on the rats were approved by the local Institutional Animal Care and Use Committee and were in accordance with the NIH Guide for the Care and Use of Laboratory Animals (2013). A total of six male Lewis rats weighing between 125 and 149 g were purchased from Envigo (Alice, TX, United States). The rats were individually housed, and for approximately 2 weeks were allowed unrestricted access to food and water. After this, food was restricted to 12–15 g per day, but water was freely available.

### Apparatus

A total of six operant conditioning chambers were used (Gerbrands, Alderston, MA, United States), each equipped with a house light overhead, three response levers, three lever lights (one above each lever), a dipper mechanism capable of delivering 0.1 ml of ethanol solution, and a pellet magazine capable of delivering 45 mg food pellets. Each chamber was housed in a light and sound-attenuating cubicle (Gerbrands). The dipper mechanism was directly opposite the ethanol-associated lever, and the pellet magazine was directly opposite the food-associated lever. The third lever was located between the food magazine and the dipper mechanism and was not used in this experiment. Chambers were interfaced with an IBM-PC compatible computer. Commercially available software was programmed to coordinate light presentations, deliver reinforcers, and record lever responses (MedPC, MedAssociates, Georgia, VT, United States).

### Ethanol self-administration

**FIGURE 1:**
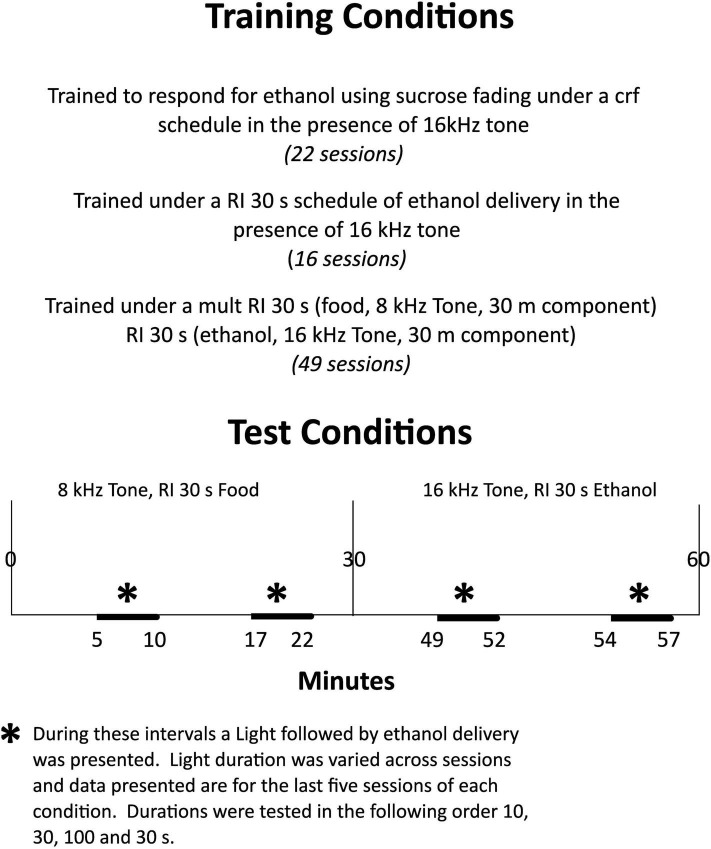
Experimental design of study. Training sequence is described in the top panel and testing conditions are presented in the lower panel.

Ethanol drinking was induced over twenty-two sessions by giving rats access to 4s presentations of 0.1 ml 8% (w/v) ethanol, and 8% sucrose solution under a continuous reinforcement schedule (CRF). Under the CRF schedule each lever press when the 80 dB, 16 kHz tone sounded produced a 4-s dipper presentation and turned off the tone. Following the dipper presentation, the tone again sounded and lever presses were reinforced. Sessions lasted 4–5 h until lever pressing occurred reliably. This took from 8 to 16 sessions. Over the remaining sessions, the sucrose concentration was reduced to zero and the session length was reduced to 1 h. The complete training sequence is illustrated in Figure 1.

Following induction of ethanol drinking and the training of responding to ethanol, rats were trained to respond to ethanol in sessions in which each response on the lever was reinforced for 15 sessions. Over the first five sessions, the schedule was moved from every response being reinforced to a random interval (RI) 30 s schedule at which value it remained for the remaining 10 sessions. The sessions were 30 min long.

After training on the RI 30s schedule of ethanol presentation, rats were placed on a multiple schedule food delivery and ethanol presentation. Responding for food was reinforced on the lever opposite the food magazine and was signaled by an 8 kHz, 80 dB tone and each delivery of a 45-mg food pellet was accompanied by a 4s timeout during which the tone was turned off. The 30min food component preceded the 30min ethanol component. The tones were present for the duration of each session, except during post-delivery timeout periods as indicated. Over 16 sessions, the schedule for food presentation was changed from one in which every lever press was reinforced to an RI 30s schedule. The schedule for ethanol presentation remained at RI 30 s throughout this time.

After 49 sessions under this mult RI 30 s (food), RI 30s (ethanol) schedule, the stimulus light above the ethanol-lever was programmed to be illuminated twice for 10 s in each component. The timing of light illumination was random, but occurred between 5 and 11 min into the food component and again between 17 and 23 min into the food component. It occurred first in the ethanol component 19–22 min into the component and then again 24–27 min into the component. Each light illumination was followed immediately by a 4s ethanol presentation. As food-responding was at relatively high constant rates throughout the food component, light illuminations occurred when responding was at high levels in this component. As ethanol-responding declined over the duration of the ethanol component, light illuminations occurred when this responding was at relatively low levels. This condition was in effect for 83 sessions. Following this, the duration of the light illumination was increased to 30 s for 20 sessions and then to 100 s for 21 sessions, and then finally returned to 30s illuminations for 47 sessions. The testing sequence is illustrated in Figure 1.

### Analysis

The main variable of analytic interest was the rate of responding during the light CS presentation compared to the period of the same duration preceding the light presentation during the last five sessions of each condition. Thus, the rate of responding for each across these sessions was calculated by dividing the number of responses during each period by the duration (s) of each period, excluding the time when the dipper was presented. These data were calculated as responses/s. The difference between response rates during CS presentation and the matched period before CS presentation is thus the primary measure.

All analyses were performed using the R statistical program (R Core Team, 2022). Comparisons were made using a repeated measures analysis of variance (ANOVA) test of a linear mixed regression using the lme:nlme and anova:r-base packages (Pinheiro et al., 2022). Changes in response rates on each lever between periods where CS was present or absent were compared, with CS duration (10 s, 30 s, 100 s) and session number (1–5) as factors. Effects with *p* < 0.05 were considered significant and further analyzed utilizing pairwise comparisons performed with multiple *t*-tests corrected using the method of Benjamini and Hochberg (1995).

An ANOVA was performed as described above to compare changes in response rates when the CS duration was 30 s with replicate (1–2) as the factor during the last five sessions of each condition. Significant main effects and interactions were further analyzed using *t*-tests and again corrected for multiple comparisons using the method of Benjamin and Hochberg. Finally, an ANOVA was performed as described above on ethanol response rates in the food components and on food response rates in the ethanol components with CS duration (10 s, 30 s, and 100 s) and session number (1–5) as factors.

## Results

**FIGURE 2:**
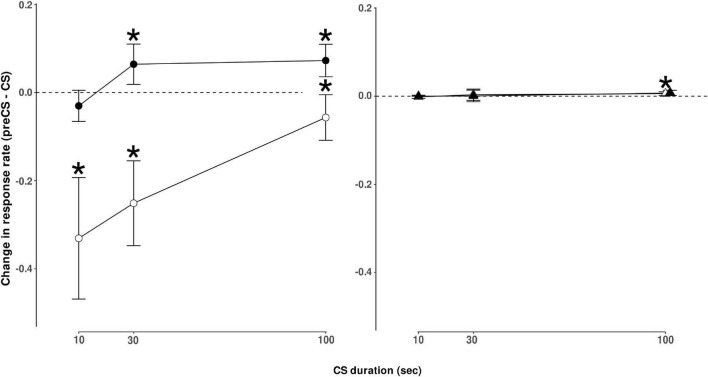
Left panel–On-target responding. Change in responding on the ethanol-lever during components where ethanol was available (•) or change in responding on the food-lever when food was available (◌) during CS presentation. Results are presented as the number of responses observed during CS presentation minus responses observed during the period immediately before CS presentation. This measure reflects the relative increase or decrease in responding during CS presentation of varying durations. CS presentation duration varied as indicated, 10-s, 30-s, or 100-s. Points represent mean change ± S.E.M. for *n* = 6 rats. *Indicates points that differ significantly from zero (no change), *p* < 0.05 after correction for multiple comparisons. Right panel–Off-target responding. Change in responding on the ethanol-lever during components where food was available (▲) or on the food-lever when ethanol was available (Δ). Results are reported as responses observed during CS presentation minus responses observed during the period immediately before CS presentation. CS presentation duration varied as indicated, 10-s, 30-s, or 100-s. Points represent mean change ± S.E.M. for *n* = 6 rats. Points above 100-s CS presentation have been adjusted to show that responding for food was significantly increased (*p* < 0.05) in the ethanol component, but not responding for ethanol in the food component.

Food-responding was suppressed more at shorter ethanol-paired CS durations than longer CS durations. As shown in Figure 2 (open circles), shorter CS presentation duration resulted in a greater decrease in response rate compared to the response rate in the period immediately preceding CS presentation, and this change diminished as CS duration increased. This was evident from the positive slope of the linear regression on change in the food response rate as a function of CS duration (mean slope [95% CI] = 3.00 × 10^–3^ [1.92 × 10^–3^ − 4.04 × 10^–3^]). An ANOVA on food response rates during food components with CS duration and session number as factors yielded a main effect of CS duration (*F*_[2,70]_ = 5.49, *p* = 0.0060). A complete ANOVA table is shown in Table 1. *Post hoc* analyses revealed that changes in food response rate from the period before stimulus presentation to the period of stimulus presentation were significant (*p* < 0.05 after correction for multiple comparisons) for all three CS durations tested, though, as noted above, the magnitude of the change decreased as a function of CS duration.

**TABLE 1:** ANOVA results for the effect of CS presentation duration and session number on responding for ethanol or food in each component type.

	Food component					Ethanol component				
		Numerator DF	Denominator DF	*F*-value	*p*-value		Numerator DF	Denominator DF	*F*-value	*p*-value
Food responses	(Intercept)	1	70	16.010	0.000	(Intercept)	1	70	0	1
	Conditioned stimulus duration	2	**70**	**5.499**	**0.006**	Conditioned stimulus duration	**2**	**70**	**4.737**	**0.012**
	Session	4	70	0.833	0.509	Session	4	70	0.249	0.909
	Conditioned stimulus × Session	8	70	0.365	0.936	Conditioned stimulus × Session	8	70	1.938	0.068
Ethanol responses	(Intercept)	1	70	0	1	(Intercept)	1	70	4.976	0.029
	Conditioned stimulus duration	2	70	1.129	0.329	Conditioned stimulus duration	**2**	**70**	**3.690**	**0.030**
	Session	4	70	0	1	Session	4	70	1.003	0.412
	Conditioned stimulus × Session	8	70	0.740	0.656	Conditioned stimulus × Session	8	70	0.813	0.594

Bolded values are those that are significant (excluding intercept values which simply imply mean is not equal to zero).

In contrast, ethanol-responding was facilitated more at longer CS durations than at shorter CS durations. As shown in Figure 2 (closed circles), no change in response rate was observed during the 10s CS presentation, compared with the period immediately prior to CS presentation, but 30 and 100s CS presentations resulted in elevated response rates compared with responding before CS presentation. This was evident from the positive slope of the linear regression on change in ethanol response rate as a function of CS duration (mean slope [95% CI] = 8.75 × 10^–4^ [3.33 × 10^–4^ − 1.42 × 10^–3^]). ANOVA on ethanol response rates during ethanol components yielded a main effect of CS duration (*F*_[2,70]_ = 3.68, *p* = 0.0300). Changes in ethanol response rates were significant when the CS duration was 30 s or 100 s (see Figure 2). The session number was not a significant factor for response rate changes for food or ethanol.

To assess the replicability of the effects observed, separate ANOVA analyses were performed on food or ethanol response rates with the two replications of the 30s CS presentation conditions as the factor. In neither case did the change in response rate upon CS presentation depend on the replicate (*F*_[1,53]_ = 0.66 and *F*_[1,53]_ = 1.43, *p* > 0.05 for ethanol and food responses, respectively).

Off-target responding (e.g., food-responding during the ethanol component) was extremely low compared with on-target responding during each component (see Table 2), but tended to increase more during longer CS durations than shorter CS durations. However, this was only reliable during the ethanol component. No effect of CS duration or session was present for changes in ethanol response rate during food components. An effect of CS duration was significant for food responses in ethanol components (*F*_[2,70]_ = 4.7, *p* < 0.05). *Post hoc* comparisons revealed that a CS duration of 100 s resulted in a significant (*p* < 0.05) increase in food-lever responding during the ethanol component. No significant effect of session number was observed, nor was there an interaction between CS duration and session number in either analysis (see Figure 2).

**TABLE 2:** Response rates (resp/sec) before (Pre-CS) and during (CS) CS presentation on each lever during each component.

	10-s								30-s								100-s							
	Food component				Ethanol component				Food component				Ethanol component				Food component				Ethanol component			
	Food		Ethanol		Ethanol		Food		Food		Ethanol		Ethanol		Food		Food		Ethanol		Ethanol		Food	
Rat	Pre-CS	CS	Pre-CS	CS	Pre-CS	CS	Pre-CS	CS	Pre-CS	CS	Pre-CS	CS	Pre-CS	CS	Pre-CS	CS	Pre-CS	CS	Pre-CS	CS	Pre-CS	CS	Pre-CS	CS
1	0.37	0.26	0.00	0.00	0.04	0.02	0.00	0.00	0.41	0.37	0.00	0.00	0.03	0.15	0.00	0.01	0.33	0.30	0.00	0.00	0.02	0.15	0.00	0.00
2	0.74	0.05	0.00	0.00	0.13	0.05	0.00	0.00	0.88	0.51	0.00	0.00	0.45	0.59	0.01	0.03	0.70	0.51	0.00	0.01	0.21	0.37	0.00	0.02
3	0.32	0.04	0.00	0.00	0.04	0.01	0.00	0.00	0.36	0.23	0.02	0.03	0.05	0.06	0.00	0.00	0.29	0.29	0.01	0.01	0.03	0.04	0.00	0.00
4	0.41	0.04	0.00	0.00	0.03	0.03	0.00	0.00	0.81	0.32	0.01	0.03	0.09	0.17	0.02	0.00	0.83	0.65	0.00	0.03	0.06	0.11	0.01	0.01
5	0.21	0.31	0.00	0.00	0.10	0.03	0.00	0.00	0.58	0.62	0.00	0.00	0.07	0.14	0.00	0.00	0.44	0.44	0.00	0.00	0.03	0.08	0.00	0.00
6	0.87	0.23	0.00	0.00	0.04	0.05	0.01	0.00	0.98	0.62	0.02	0.02	0.03	0.08	0.00	0.01	0.55	0.61	0.02	0.02	0.02	0.05	0.00	0.01
Mean	0.49	0.16	0.00	0.00	0.06	0.03	0.00	0.00	0.67	0.45	0.01	0.01	0.12	0.20	0.01	0.01	0.52	0.47	0.01	0.01	0.06	0.13	0.00	0.01
S.E.M.	0.10	0.05	0.00	0.00	0.02	0.01	0.00	0.00	0.11	0.07	0.00	0.01	0.07	0.08	0.00	0.00	0.09	0.06	0.00	0.00	0.03	0.05	0.00	0.00

## Discussion

Here we report that an ethanol-paired CS can enhance ethanol-maintained responding and simultaneously disrupt food-maintained responding. The effect on ethanol-maintained responding is most pronounced at longer CS presentations, while the effect on food-maintained responding decreases as a function of CS length. This observation is consistent with previous studies showing that ethanol-paired stimuli can increase ethanol-seeking, perhaps by increasing craving or motivation to consume ethanol (Krank, 2003; e.g., Corbit and Janak, 2007; Lamb et al., 2016a). This observation is also consistent with the phenomenon of positive conditioned suppression whereby a CS associated with a desirable event can reduce engagement in other activities (Azrin and Hake, 1969; Miczek and Grossman, 1971). Further, these observations are consistent with the change in operant behavior occurring because of the CR elicited by the CS, which depends upon the form of the CS, and its duration (Holland, 1977, 1980a,b). The implication of this work is that the effect of alcohol-paired CS presentation may have differential effects on ethanol-seeking or alternative behavior, depending on the duration, form, and timing of CS exposure.

Ethanol-paired-stimuli increase ethanol-responding and decrease food-responding (Krank, 2003; Corbit and Janak, 2007; Lamb et al., 2016a,^2020^, this study). These outcomes are consistent with the idea that ethanol-paired-stimuli increase craving, which in turn is a result of increased motivation to drink ethanol, or more simply ethanol desire (Pomerleau et al., 1983). This increased ethanol desire may distract from the performance of other behavior. Both the increase in ethanol desire and the disruption of other behavior might be expected to promote excessive drinking and relapse (Lamb et al., 2016b; Lamb and Ginsburg, 2018). The procedure demonstrated here provides a means of examining both ethanol-paired-stimuli-induced increases in ethanol-seeking and ethanol-paired-stimuli-induced disruption of other behavior; and to the extent that these reflect craving, a means for studying craving using a steady-state procedure.

As already mentioned, craving is generally thought to result in decreases in other behavior and increases in ethanol-seeking (Lamb and Ginsburg, 2018; Bowen et al., 2022). However, it is equally possible that these two behaviors are what result in craving, i.e., that upon observing that one’s routine behavior is disrupted by things that might signal opportunities to drink or that one is seeking ethanol, especially during recovery, when drinking is suppressed or unavailable, one learns this phenomenon called “craving a drink.” We favor the latter viewpoint. Craving is a subjective effect, descriptive of a situation that cannot be objectively observed or measured, and subject to variability in meaning and reporting across individuals or social or cultural groups (Angel and Gronfein, 1988). Thus, one comes to use the term craving in situations and feelings associated with an increased likelihood of drinking or having one’s behavior disrupted by thoughts of drinking and noting when you might seek alcohol rather than other activities, as this usage is reinforced by those around you (see Lamb et al., 1991; Lamb and Henningfield, 1994). If craving causes disruptions in other behavior and an increased propensity to drink, then measures of behavioral disruption and drinking are likely to be less sensitive than measures of craving. On the other hand, if behavioral disruption and an increased propensity to drink occasion reports of craving, behavioral disruption and propensity to drink are likely to be more sensitive measures. So far in other similar situations, direct behavioral measures have been more sensitive measures than subjective effects, e.g., lower doses of morphine occasion drug-seeking than those needed to occasion reports of drug liking (Lamb et al., 1991).

Still, that craving reflects increased ethanol desire could be true no matter if it is a case of increased ethanol desire causing craving, which disrupts other behavior and increases drinking, or if increased ethanol desire increases ethanol-seeking and disrupts other behavior, which occasion reports of craving. However, the results of the present experiment are not consistent with the idea that ethanol-paired-stimuli increase ethanol-seeking *and* disrupt other behavior by increasing ethanol desire either directly or through an increase in craving. If increased ethanol desire was responsible for *both* the increase in ethanol-seeking and the disruption of other behavior then *both* should increase as ethanol desire increases. However, increasing CS duration increases ethanol-seeking, while decreasing the disruption of other behavior. Conversely, decreasing the CS duration increases disruption of other behavior, while attenuating the increase in ethanol-seeking. These observations are inconsistent with increases in ethanol-seeking *and* disruptions of other behavior seen during the presentation of ethanol-paired stimuli *both* being direct consequences of ethanol-paired-stimulus-elicited increases in ethanol desire.

These observations are more consistent with the change in operant behavior seen following CS presentation being a consequence of the CR elicited by the CS, which will depend upon the form of the CS and its duration (Holland, 1977, 1980a,b). Short CS frequently elicits orienting responses. In the case of food-responding in the present experiment, this involves looking and perhaps moving away from the food-lever. In the case of ethanol-responding, the ethanol-paired-stimulus was immediately above the ethanol-lever. Food-responding was decreased by the shorter CS, while ethanol-responding was essentially unaffected by the short CS. Results consistent with this hypothesis can be seen in experiments in which the CS location is varied. Karpicke et al. (1977) found suppression of food-responding when the CS was located away from the food-lever, but little effect of the CS on food-responding when the CS was located near the food-lever. Particularly germane to this argument, Krank et al. (2008) found that ethanol-paired-stimuli attracted approach and when these were located near the ethanol-lever, ethanol-paired-stimuli increased ethanol-responding. Such arguments are consistent with the roles of CS in drug addiction postulated by Tomie (1996) and Flagel et al. (2009) in which the attractive properties of the CS when appropriately situated help promote further drug-taking and addiction.

Conditioned stimuli not only elicit behavior that might promote addiction, CS also provide information. In the case of this experiment, the CS foretold the delivery of ethanol above and beyond that ordinarily available. This increased density of ethanol reinforcement might be expected to increase ethanol-responding under a random interval schedule, and to exert less effect on food-responding, with the increases resulting from generalization from the ethanol-lever to the food-lever or decreases resulting from rats responding on the ethanol-lever rather than the food-lever. It should be noted that stimulus control in this experiment was excellent and very few off-target responses were observed either in the presence or absence of the CS (though there were slightly more during the CS). The effect of a signaled increase in ethanol reinforcement density and the cue light approach elicited by the ethanol-paired-stimulus are likely in conflict. Thus, it is not surprising that increases in ethanol-responding are most readily observed at longer CS durations that appear to elicit fewer incompatible CRs.

In this study, rats were food-restricted. This allowed us to use food-maintained behavior as a comparison to ethanol-maintained behavior to determine the specificity of CS effects. While this condition may have affected our results, it is important to note that others have seen similar ethanol-associated CS effects on responding for ethanol in rats with no food restriction (Corbit and Janak, 2007) as well as food-restricted rats (Lamb et al., 2020). Additionally, others have shown that longer duration food-associated CS can increase food-maintained behavior in food-restricted animals (Meltzer and Brahlek, 1970; Miczek and Grossman, 1971), and when the CS-duration is shorter decreases in food-maintained behavior have been observed (Azrin and Hake, 1969). Further, in the present study, an ethanol-associated CS increased ethanol-maintained responding and not food-maintained responding in food-restricted rats (Figure 2). Thus, it is unlikely that these results are dependent on the food-restriction status of the subjects.

Conditioned stimuli are thought to play a role in relapse to alcohol or drug use disorders by producing craving, which is thought to reflect an increased desire for alcohol or drug. Craving, in turn, is thought to result in increased drug-seeking and the disruption of other ongoing behavior. While both outcomes upon exposure to an ethanol-associated CS might be considered “craving,” they do not appear to occur solely as the result of increased ethanol motivation, or else they should co-vary. Instead, these results show that increased ethanol-responding and decreased food-responding can occur under different CS presentation conditions, suggesting other mechanisms beyond motivation for alcohol are involved. Specifically, ethanol-paired CS presentation increases responding to ethanol, and this effect *increases* with CS presentation duration. In contrast, CS presentation decreases responding to food, and this effect *decreases* with CS presentation duration. This outcome is inconsistent with an account of CS increasing drug-seeking and decreasing other ongoing behavior due to increased motivation for the CS-paired drug, due to the contrasting effect of longer CS presentation on ethanol-maintained and food-maintained behavior. This outcome is consistent with an informational account of CS action on drug-seeking, whereby the CS indicates a likely increase in the density of the paired US (ethanol), while eliciting behavior toward the paired stimulus or US (ethanol)-delivery location that is incompatible with behavior maintained by the unpaired US (food) at shorter durations. These findings have two important implications for how craving might best be conceptualized. First, as it is commonly used craving refers both to an increased likelihood of future drug use and to a disruption of ongoing behavior resulting from stimuli and situations associated with past drug use. In this case, our results indicate craving likely represents multiple different behavioral processes, not simply increased motivation. Second, these results provide further evidence that the use of the term craving is likely as a subjective effect representing an assessment based upon past experiences, rather than reports about a causal mechanism that changes the likelihood of future behavior.

## Data availability statement

The raw data supporting the conclusions of this article will be made available by the authors, without undue reservation.

## Ethics statement

The animal study was reviewed and approved by Institutional Animal Use and Care Committee, UT Health Science Center at San Antonio.

## Author contributions

BG was involved in the analysis and authorship of this work. AN-M was involved in the analysis and visualization of the results. CS and RL were involved in study design, interpretation, and authorship. All authors contributed to the article and approved the submitted version.

## References

[B1] Angel R. Gronfein W. (1988). The use of subjective information in statistical models. *Am. Sociol. Rev.* 53 464–473. 10.2307/2095653

[B2] Azrin N. H. Hake D. F. (1969). Positive conditioned suppression: conditioned suppression using positive reinforcers as the unconditioned stimuli1. *J. Exp. Anal. Behav.* 12 167–173. 10.1901/jeab.1969.12-167 16811337 PMC1338587

[B3] Benjamini Y. Hochberg Y. (1995). Controlling the false discovery rate: a practical and powerful approach to multiple testing. *J. R. Stat. Soc. Ser. B Methodol.* 57 289–300.

[B4] Bowen M. T. George O. Muskiewicz D. E. Hall F. S. (2022). Factors contributing to the escalation of alcohol consumption. *Neurosci. Biobehav. Rev.* 132 730–756. 10.1016/j.neubiorev.2021.11.017 34839930 PMC8892842

[B5] Corbit L. H. Janak P. H. (2007). Ethanol-Associated cues produce general pavlovian-instrumental transfer. *Alcohol. Clin. Exp. Res.* 31 766–774. 10.1111/j.1530-0277.2007.00359.x 17378919

[B6] Corbit L. H. Janak P. H. (2016). Habitual alcohol seeking: neural bases and possible relations to alcohol use disorders. *Alcohol. Clin. Exp. Res.* 40 1380–1389. 10.1111/acer.13094 27223341 PMC5600324

[B7] Duncan P. M. (1997). Conditioned suppression of operant responding in response to a stimulus paired with pentobarbital injections. *Psychobiology* 25 146–151. 10.3758/BF03331920

[B8] Duncan P. M. Barry T. Ellis R. Hinkle E. (1989). Conditioned response to amphetamine injection with the operant paradigm. *Drug Dev. Res.* 16 133–141. 10.1002/ddr.430160207

[B9] Estes W. K. (1943). Discriminative conditioning. i. a discriminative property of conditioned anticipation. *J. Exp. Psychol.* 32 150–155. 10.1037/h0058316

[B10] Estes W. K. Skinner B. F. (1941). Some quantitative properties of anxiety. *J. Exp. Psychol.* 29 390–400. 10.1037/h0062283

[B11] Flagel S. B. Akil H. Robinson T. E. (2009). Individual differences in the attribution of incentive salience to reward-related cues: implications for addiction. *Neuropharmacology* 56 139–148. 10.1016/j.neuropharm.2008.06.027 18619474 PMC2635343

[B12] Henton W. W. Brady J. V. (1970). Operant acceleration during a pre-reward stimulus1. *J. Exp. Anal. Behav.* 13 205–209. 10.1901/jeab.1970.13-205 16811437 PMC1333762

[B13] Holland P. C. (1977). Conditioned stimulus as a determinant of the form of the Pavlovian conditioned response. *J. Exp. Psychol. Anim. Behav. Process.* 3 77–104. 10.1037/0097-7403.3.1.77 845545

[B14] Holland P. C. (1980a). CS-US interval as a determinant of the form of Pavlovian appetitive conditioned responses. *J. Exp. Psychol. Anim. Behav. Process.* 6 155–174. 10.1037/0097-7403.6.2.155 7373230

[B15] Holland P. C. (1980b). Influence of visual conditioned stimulus characteristics on the form of Pavlovian appetitive conditioned responding in rats. *J. Exp. Psychol. Anim. Behav. Process.* 6 81–97. 10.1037/0097-7403.6.1.81 7373228

[B16] Hunt H. F. Brady J. V. (1951). Some effects of electro-convulsive shock on a conditioned emotional response (“anxiety”). *J. Comp. Physiol. Psychol.* 44 88–98. 10.1037/h0059967 14814246

[B17] Karpicke J. Christoph G. Peterson G. Hearst E. (1977). Signal location and positive versus negative conditioned suppression in the rat. *J. Exp. Psychol. Anim. Behav. Process.* 3 105–118. 10.1037/0097-7403.3.2.105

[B18] Krank M. D. (2003). Pavlovian conditioning with ethanol: sign-tracking (autoshaping), conditioned incentive, and ethanol self-administration. *Alcohol. Clin. Exp. Res.* 27 1592–1598. 10.1097/01.ALC.0000092060.09228.DE 14574229

[B19] Krank M. D. O’Neill S. Squarey K. Jacob J. (2008). Goal- and signal-directed incentive: conditioned approach, seeking, and consumption established with unsweetened alcohol in rats. *Psychopharmacology (Berl.)* 196 397–405. 10.1007/s00213-007-0971-970 17965977

[B20] Kruzich P. J. Congleton K. M. See R. E. (2001). Conditioned reinstatement of drug-seeking behavior with a discrete compound stimulus classically conditioned with intravenous cocaine. *Behav. Neurosci.* 115 1086–1092. 10.1037//0735-7044.115.5.1086 11584921

[B21] Lamb R. J. Ginsburg B. C. (2018). Addiction as a BAD, a behavioral allocation disorder. *Pharmacol. Biochem. Behav.* 164 62–70. 10.1016/j.pbb.2017.05.002 28476485 PMC6089073

[B22] Lamb R. J. Ginsburg B. C. Greig A. Schindler C. W. (2019). Effects of rat strain and method of inducing ethanol drinking on Pavlovian-Instrumental-Transfer with ethanol-paired conditioned stimuli. *Alcohol* 79 47–57. 10.1016/j.alcohol.2019.01.003 30641121 PMC7201463

[B23] Lamb R. J. Ginsburg B. C. Schindler C. W. (2016a). Effects of an ethanol-paired CS on responding for ethanol and food: comparisons with a stimulus in a truly-random-control group and to a food-paired CS on responding for food. *Alcohol Fayettev. N* 57 15–27. 10.1016/j.alcohol.2016.10.009 27916139 PMC6089076

[B24] Lamb R. J. Maguire D. R. Ginsburg B. C. Pinkston J. W. France C. P. (2016b). Determinants of choice, and vulnerability and recovery in addiction. *Behav. Processes* 127 35–42. 10.1016/j.beproc.2016.04.001 27083500 PMC4968700

[B25] Lamb R. J. Schindler C. W. Pinkston J. W. (2016c). Conditioned stimuli’s role in relapse: preclinical research on Pavlovian-Instrumental-Transfer. *Psychopharmacology (Berl.)* 233 1933–1944. 10.1007/s00213-016-4216-y 26800688 PMC4863941

[B26] Lamb R. J. Ginsburg B. C. Schindler C. W. (2017). Conditioned stimulus form does not explain failures to see pavlovian-instrumental-transfer with ethanol-paired conditioned stimuli. *Alcohol. Clin. Exp. Res.* 41 1063–1071. 10.1111/acer.13376 28294355 PMC5542406

[B27] Lamb R. J. Henningfield J. E. (1994). Human d-amphetamine drug discrimination: methamphetamine and hydromorphone. *J. Exp. Anal. Behav.* 61 169–180. 10.1901/jeab.1994.61-169 7513346 PMC1334405

[B28] Lamb R. J. Preston K. L. Schindler C. W. Meisch R. A. Davis F. Katz J. L. (1991). The reinforcing and subjective effects of morphine in post-addicts: a dose-response study. *J. Pharmacol. Exp. Ther.* 259 1165–1173. 1762068

[B29] Lamb R. J. Schindler C. W. Ginsburg B. C. (2020). Ethanol-paired stimuli can increase reinforced ethanol responding. *Alcohol* 85 27–34. 10.1016/j.alcohol.2019.10.007 31689483 PMC7195246

[B30] Lovibond P. F. (1983). Facilitation of instrumental behavior by a Pavlovian appetitive conditioned stimulus. *J. Exp. Psychol. Anim. Behav. Process.* 9 225–247. 10.1037/0097-7403.9.3.225 6153052

[B31] Meltzer D. Brahlek J. A. (1970). Conditioned suppression and conditioned enhancement with the same positive UCS: an effect of CS duration. *J. Exp. Anal. Behav.* 13:67. 10.1901/jeab.1970.13-67 5415041 PMC1333658

[B32] Miczek K. A. Grossman S. P. (1971). Positive conditioned suppression: effects of Cs duration1. *J. Exp. Anal. Behav.* 15 243–247. 10.1901/jeab.1971.15-243 16811509 PMC1333809

[B33] Pinheiro J. Bates D. DebRoy S. Sarkar D. R Core Team (2022). *nlme: Linear and Nonlinear Mixed Effects Models.* Springe: New York. 10.1007/b98882

[B34] Pomerleau O. F. Fertig J. Baker L. Cooney N. (1983). Reactivity to alcohol cues in alcoholics and non-alcoholics: implications for a stimulus control analysis of drinking. *Addict. Behav.* 8 1–10. 10.1016/0306-4603(83)90048-90045 6880920

[B35] R Core Team (2022). *R: A Language and Environment for Statistical Computing.* Vienna: R Foundation for Statistical Computing.

[B36] Schindler C. W. Thorndike E. B. Ma J. D. Goldberg S. R. (2000). Conditioned suppression with cocaine as the unconditioned stimulus. *Pharmacol. Biochem. Behav.* 65 83–89. 10.1016/S0091-3057(99)00176-178 10638640

[B37] Stein L. Sidman M. Brady J. V. (1958). Some effects of two temporal variables on conditioned suppression. *J. Exp. Anal. Behav.* 1 153–162. 10.1901/jeab.1958.1-153 16811211 PMC1403932

[B38] Tiffany S. T. (1990). A cognitive model of drug urges and drug-use behavior: role of automatic and nonautomatic processes. *Psychol. Rev.* 97 147–168. 10.1037/0033-295X.97.2.147 2186423

[B39] Tomie A. (1996). Locating reward cue at response manipulandum (CAM) induces symptoms of drug abuse. *Neurosci. Biobehav. Rev.* 20 505–535. 10.1016/0149-7634(95)00023-22 8880737

[B40] Waller M. B. Waller P. F. (1963). The effects of unavoidable shocks on a multiple schedule having an avoidance component1. *J. Exp. Anal. Behav.* 6 29–37. 10.1901/jeab.1963.6-29 13998594 PMC1404236

[B41] Watanabe S. (1990). isodirectional conditioning effects of d-amphetamine and pentobarbital on schedule-controlled operant behavior in pigeons. *Pharmacol. Biochem. Behav.* 36 157–161. 10.1016/0091-3057(90)90142-90145 2349257

